# NonTuberculous Mycobacteria infection and lung transplantation in cystic fibrosis: a worldwide survey of clinical practice

**DOI:** 10.1186/s12890-018-0635-3

**Published:** 2018-05-22

**Authors:** Adrien Tissot, Matthew F. Thomas, Paul A. Corris, Malcolm Brodlie

**Affiliations:** 1 Institute of Cellular Medicine, Newcastle University, Medical School, Framlington Place, Newcastle upon Tyne, NE2 4HH UK; 2 Institute of Transplantation, Freeman Hospital, Newcastle upon Tyne Hospitals NHS Foundation Trust, Newcastle upon Tyne, NE7 7DN UK; 3 Centre Hospitalier Universitaire de Nantes, Nantes, France; 4 Great North Children’s Hospital, Newcastle upon Tyne Hospitals NHS Foundation Trust, Level 3, Clinical Resource Building, Queen Victoria Road, Newcastle upon Tyne, NE1 4LP UK

**Keywords:** Cystic fibrosis, Nontuberculous mycobacteria, Lung transplantation, *Mycobacterium abscessus*, *Mycobacterium avium*

## Abstract

**Background:**

In people with cystic fibrosis infection with NonTuberculous Mycobacteria is of increasing prevalence. *Mycobacterium abscessus* complex is of particular concern and has been associated with adverse clinical outcomes. Optimal treatment usually requires multiple antibiotics for over 12 months. When considering lung transplantation for patients with NonTuberculous Mycobacteria potential benefits must be balanced against the risks of uncontrolled infection post-transplant and significant side-effects associated with treatment. In this survey we assessed current international practice with regard to assessing and listing patients for lung transplantation.

**Methods:**

We designed a questionnaire enquiring about local practice regarding screening for NonTuberculous Mycobacteria infection, specific contra-indications to transplantation, management and segregation of patients pre- and post-transplant. The survey was sent via e-mail to 37 paediatric and adult lung transplant centres across Europe, North America and Australia.

**Results:**

We gathered complete questionnaires from 21 centres (57% response rate). Few centres (29%) have a clear written policy regarding NonTuberculous Mycobacteria. Sixteen (76%) centres require molecular identification of NonTuberculous Mycobacteria species. Only four centres would consider infection with *M. abscessus* complex in itself a contra-indication for listing, however 76% regard it as a relative contra-indication. Eighty-six percent require treatment pre-transplantation. Finally, only 61% of centres had a clear policy regarding segration of patients pre-transplant and 48% post-transplant.

**Conclusions:**

The issue of NonTuberculous Mycobacteria infection in people with cystic fibrosis requiring lung transplantation is well-recognized however current international recommendations are not detailed and there is variation in practice between centres. There is an urgent requirement for high quality clinical data to inform decision-making.

## Background

Pulmonary infection with nontuberculous mycobacteria (NTM) in people with cystic fibrosis (CF) is of increasing importance and concern to both patients and clinicians [1]. Infection with NTM is of particular relevance during the assessment process of suitability for lung transplantation [2].

The incidence of respiratory cultures positive for NTM in people with CF has risen over recent decades [3]. Multicenter studies have reported varying prevalence that ranges from 2.7% across Europe [4], 6.6% in France [5] to 14% throughout the United States (US) [6]. Significant geographical variation has also been identified, for example in the US some states have been found to have a prevalence as high as 20% whereas in others it is much lower at < 5% [6]. Environmental factors are thought to influence this variation but screening practices may also vary between individual CF centres. The most common NTM species isolated from people with CF are *Mycobacterium abscessus* complex and *M. avium* complex (MAC).

The American Thoracic Society/Infectious Disease Society of America definition of NTM pulmonary disease (NTM PD) involves clinical, radiological and microbiological criteria and is widely used clinically [7]. Importantly, pulmonary infection with *M. abscessus* complex in people with CF has been associated with accelerated decline in lung function and is of particular clinical concern [4, 8–10]. There is also emerging evidence to suggest transmission of *M. abscessus* complex between individuals with CF [11, 12]. NTM PD is challenging to treat requiring multiple antibiotic regimes for a prolonged period of at least 12 months [13, 14]. The antibiotics used are frequently associated with adverse effects and represent a significant treatment burden for patients. Ultimately NTM are also often difficult to eradicate from the airways of people with CF despite treatment.

In the past pulmonary NTM infection in patients with CF was considered an absolute contra-indication for lung transplantation by some groups due to poor post-transplant outcomes that were largely attributed to uncontrolled infection in the context of systemic immunosuppression, though the ISHLT guidelines never endorsed NTM infection as an absolute contraindication [15–17]. Soft tissue and wound infections are often particularly challenging to treat post-operatively [18]. Specifically, the presence of *M. abscessus* complex pre-transplant has been associated with severe clinical problems and poor outcomes following lung transplantation [15–17]. The specific subspecies of *M. abscessus* complex may also impact on risk stratification [19]. The isolation of NTM in respiratory samples post-lung transplantation has also been associated with adverse clinical outcomes [18, 20].

However, some single-centre case series have suggested that lung transplantation in people with NTM infection can be associated with comparable outcomes to those free of NTM infection [21–23]. Albeit with the caveat that anti-NTM treatment is likely to need to be prolonged and may be associated with adverse effects and complications.

The 2014 update for the selection of lung transplant candidates from the International Society for Heart and Lung Transplantation (ISHLT) recommends *“controlled chronic infection pre-operatively and a reasonable expectation for adequate control post-operatively”* in order for patients with NTM to be safely listed for transplantation [24]. Recent consensus recommendations from the United States Cystic Fibrosis Foundation (CFF) and the European Cystic Fibrosis Society (ECFS) emphasize the need to thoroughly evaluate patients with CF for NTM as part of the lung transplant assessment process and to start treatment prior to transplant listing but largely leave decision-making open to the individual centre and clinician [14].

The increasing prevalence of NTM in people with CF means that it is becoming more and more of an issue during the process of assessment of suitability for lung transplantation [2]. Anecdotally we have observed different approaches in the global lung transplantation community towards this challenging clinical area and we therefore wished to investigate current practice worldwide with regard to assessing and listing patients for lung transplantation with CF who have NTM infection.

## Methods

**Fig. 1:**
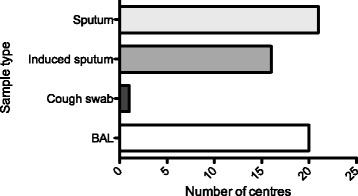
Type of respiratory samples considered as relevant for NTM screening at assessment for lung transplantation. Total number of centres returning questionnaires = 21. Answers to the question: *“Do you consider the following respiratory samples as relevant for NTM screening?”.* BAL: bronchio-alveolar lavage

We designed a survey consisting of 16 questions enquiring about local practice regarding screening for NTM infection, specific contra-indications to transplantation, management and segregation of patients pre- and post-transplant (Fig. 1). This questionnaire was sent via e-mail to 37 adult and pediatric lung transplantation centres across Europe, North America and Australia in April 2016.

## Results

We received complete questionnaires from 21 centres (57% response rate).

### Screening for NTM during transplant assessment process

All centres specifically screened patients with CF for NTM infection pre-transplant but only a minority (29%) had a clear written policy regarding this issue (Table 1). Sixteen centres (76%) required molecular identification of NTM species. The main respiratory samples (Fig. 1) accepted as relevant were sputum and broncho-alveolar lavage (100 and 95% respectively). Induced sputum was also considered acceptable by 16 centres. Only one centre based decisions on results from cough swabs.

**Table 1:** Questionnaire results (*n* = 21 centres)

Question	Yes n (%)
Do you have a clear written policy regarding the assessment and listing of patients with CF and NTM infection?	6 (29)
During assessment do you require screening for NTM?	21 (100)
Do you require molecular identification of NTM species?	16 (76)
Do you regard current infection with MAC an absolute contraindication?	1 (5)
Do you regard current infection with *M. abscessus* complex as an absolute contraindication?	4 (19)
Do you regard persisting MAC or *M. abscessus* complex infection despite optimal therapy as an absolute contraindication?	12 (57)
Would you regard a patient with persisting *M. abscessus* complex and another relative contraindication as together representing an absolute contraindication?	16 (76)
Do you require specific NTM treatment to be undertaken before listing?	18 (86)
At time of listing is a treatment cocktail decided on for use peri-transplant?	20 (95)
Are patients segregated on the basis of NTM status pre-transplant?	13 (62)
Are patients segregated post-transplant on the basis of NTM status?	10 (48)

*Abbreviations*: CF Cystic Fibrosis, *NTM* NonTuberculous Mycobacteria, *MAC Mycobacterium avium* Complex, *M. abscessus complex Mycobacteria abscessus* complex

### Policies for suitability of patients with NTM for lung transplantation listing

**Fig. 2:**
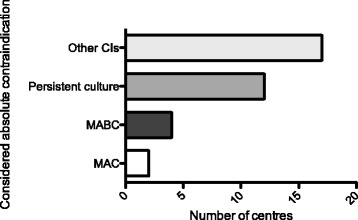
What is considered a contraindication to lung transplantation at each centre? Total number of centres returning questionnaires = 21. In answer to the question, “are the following considered an absolute contraindication for lung transplantation?”: MAC: current infection with *Mycobacterium avium* Complex; MABC: current infection with *Mycobacterium abscessus* complex; Persistent culture: persisting MAC or *M. abscessus* complex infection despite optimal therapy; other CI: Would you regard a patient with persisting *M. abscessus* complex and another relative contraindication as together representing an absolute contraindication?

Only four centers considered infection with *M. abscessus* complex in itself an absolute contra-indication for transplant listing (Fig. 2). More than half (57%) stated they would not list a patient with persisting positive respiratory cultures despite optimal treatment. A majority (76%) of centres considered persistent *M. abscessus* complex infection to be a relative contraindication to listing if other adverse factors are present. Eighteen centres require treatment pre-transplant and 20 (95%) would decide a peri-transplant treatment cocktail at the time of listing.

### Infection control policies

Finally, 13, centres (61%) apply a segregation policy based on NTM status of patients pre-transplant and 10 (48%) post-transplantation, for example in outpatient clinic waiting areas.

## Discussion

This survey captures current practice in the global lung transplant community with regard to patients with CF and NTM infection. This is well recognised to be a challenging issue clinically with a dearth of high quality evidence on which to base decision-making. Most centres do currently consider patients with CF and NTM infection for lung transplantation assessment and potential listing, i.e. it is not considered an absolute contraindication. However, the majority do consider it a relative contraindication if other comorbidities or relative contraindications are present. The specific species of NTM and response to treatment pre-transplant are also taken in to account during the decision-making process at the majority of centres.

In our questionnaire, we asked about isolation of NTM from respiratory samples, i.e. infection rather than patients who fulfil the ATS NTMPD criteria per se*.* This was because the presence alone of NTM, and *M. abscessus complex* in particular, is a significant potential concern in the context of immunosuppression post-major thoracic surgery as is the case following lung transplantation.

Our findings highlight the importance of further high-quality clinical research to comprehensively investigate the outcomes and international experience of patients with NTM after lung transplantation. Other key questions include risk stratification around specific species and subspecies, resistance profiles, response to treatment and disease-burden pre-transplant. For example, In a single centre paediatric case series adverse outcomes were associated with infection pre-transplant with *M. abscessus* subspecies *abscessus* with improved results in children with *M. abscessus* subspecies *bolletti* or *massilliense* infection [19]. *M. avium* complex is the most prevalent grouping of NTM in North American patients with CF and data is equally lacking around outcomes and best practice in individuals with *M.avium* complex infection.

The variation in international practice that we have identified affirms the current attention being given to NTM infection in respiratory disease and CF in particular. As a poorly-defined strong relative contraindication to lung transplantation NTM and *M. abscessus* complex status in particular is of huge relevance to individual patients in terms of lung transplantation listing being a viable option or not for them, which may be life-changing. Evidence to support an increasing prevalence of NTM in people with CF continues to grow. The proportion of patients affected and the complexity of treatment necessary in terms of length, toxicity and limited efficacy make infection with NTM an issue of major concern for CF clinicians. The specific question of considering lung transplantation for these patients adds a further level of complexity with the added post-transplant threat of systemic immunosuppression and increased risk of drug-related toxicity.

It is clear in both the ISHLT and the CFF/ECFS consensus recommendations that NTM infection has to be looked for and if possible treated pre-transplant. The ISHLT stated that *“chronic infection with highly virulent and/or resistant microbes that are poorly controlled pre-transplant”* is an absolute contraindication, which is a situation that can been seen in NTM infection. In the CFF/ECFS document it is recommended that *“the presence of persistent MABC or MAC infection despite optimal therapy is not an absolute contraindication for lung transplant referral”*. Pragmatically therefore many transplant clinicians firstly evaluate the “controlled” character of the chronic infection or colonization with NTM pre-transplant and then evaluate the post-transplant risk associated with the presence of NTM. It is interesting to note that almost all centres do not consider in itself the presence of NTM as an absolute contraindication but a majority would not list a patient with a failure of an optimal therapy to eradicate NTM or if associated with another relative contraindication. This illustrates in our view the central concern of clinicians to have an available efficient combination of antibiotics that can be tolerated by the patient before moving on to the difficult process of lung transplantation.

Finally, the relatively low rate of centres segregating patients depending on NTM status pre- and post-transplant is concerning when placed in the context of the recent studies suggesting potential transmission of *M. abscessus* complex between patients with CF [11, 12, 25]. Extensive and elaborate infection control measures have been recommended to prevent such transmission events [14]. The likely low number of patients post-transplant with NTM is also a possible explanation for the lower number of centres segregating post-transplant [18, 20].

There are some limitations to our work. Firstly, we did not send the questionnaire to all existing lung transplantation centres. Our objective was not to be exhaustive but to have a representative sample as in an opinion poll. Secondly, we did not adjust the results for the size of centre, which means an answer from a small centre, for example performing less than 20 transplants per year, has the same value as that from a larger centre.

## Conclusions

In conclusion, the issue of NTM infection in patients with CF requiring lung transplantation is of current extensive debate and discussion amongst the lung transplantation community. Although many clinicians anecdotally suspected sub-optimal outcomes following transplantation in patients with NTM, most centres do not consider it as an absolute contra-indication unless associated with other comorbidities or in the absence of a suitable cocktail for antimicrobial therapy. Practice is not consistent however between centres and there is an urgent requirement for the collection and analysis of high-quality clinical data followed by development of an evidence-based guideline on this specific issue.

## Abbreviations

CF: Cystic fibrosis
CFF: Cystic Fibrosis Foundation
ECFS: European Cystic Fibrosis Society
ISHLT: International Society for Heart and Lung Transplantation
MAC: *Mycobacterium avium* complex
NTM: Nontuberculous mycobacteria
NTMPD: Nontuberculous mycobacterial pulmonary disease

## References

[1] Leung JM, Olivier KN (2013). Nontuberculous mycobacteria: the changing epidemiology and treatment challenges in cystic fibrosis. Curr Opin Pulm Med.

[2] Lobo LJ, Noone PG (2014). Respiratory infections in patients with cystic fibrosis undergoing lung transplantation. Lancet Respir Med.

[3] Prevots DR, Marras TK (2015). Epidemiology of human pulmonary infection with nontuberculous mycobacteria: a review. Clin Chest Med.

[4] Martiniano SL, Sontag MK, Daley CL, Nick JA, Sagel SD (2014). Clinical significance of a first positive nontuberculous mycobacteria culture in cystic fibrosis. Ann Am Thorac Soc.

[5] Roux AL, Catherinot E, Ripoll F, Soismier N, Macheras E, Ravilly S, Bellis G, Vibet MA, Le Roux E, Lemonnier L (2009). Multicenter study of prevalence of nontuberculous mycobacteria in patients with cystic fibrosis in France. J Clin Microbiol.

[6] Adjemian J, Olivier KN, Prevots DR (2014). Nontuberculous mycobacteria among patients with cystic fibrosis in the United States: screening practices and environmental risk. Am J Respir Crit Care Med.

[7] Griffith DE, Aksamit T, Brown-Elliott BA, Catanzaro A, Daley C, Gordin F, Holland SM, Horsburgh R, Huitt G, Iademarco MF (2007). An official ATS/IDSA statement: diagnosis, treatment, and prevention of nontuberculous mycobacterial diseases. Am J Respir Crit Care Med.

[8] Qvist T, Taylor-Robinson D, Waldmann E, Olesen HV, Hansen CR, Mathiesen IH, Hoiby N, Katzenstein TL, Smyth RL, Diggle PJ (2016). Comparing the harmful effects of nontuberculous mycobacteria and Gram negative bacteria on lung function in patients with cystic fibrosis. J Cyst Fibros.

[9] Oliver A, Maiz L, Canton R, Escobar H, Baquero F, Gomez-Mampaso E (2001). Nontuberculous mycobacteria in patients with cystic fibrosis. Clin Infect Dis.

[10] Griffith DE (2014). Mycobacterium abscessus subsp abscessus lung disease: ‘trouble ahead, trouble behind...’. F1000Prime Rep.

[11] Bryant JM, Grogono DM, Greaves D, Foweraker J, Roddick I, Inns T, Reacher M, Haworth CS, Curran MD, Harris SR (2013). Whole-genome sequencing to identify transmission of Mycobacterium abscessus between patients with cystic fibrosis: a retrospective cohort study. Lancet.

[12] Bryant JM, Grogono DM, Rodriguez-Rincon D, Everall I, Brown KP, Moreno P, Verma D, Hill E, Drijkoningen J, Gilligan P (2016). Emergence and spread of a human-transmissible multidrug-resistant nontuberculous mycobacterium. Science.

[13] Skolnik K, Kirkpatrick G, Quon BS (2016). Nontuberculous mycobacteria in cystic fibrosis. Curr Treat Options Infect Dis.

[14] Floto RA, Olivier KN, Saiman L, Daley CL, Herrmann JL, Nick JA, Noone PG, Bilton D, Corris P, Gibson RL (2016). US Cystic Fibrosis Foundation and European cystic fibrosis society consensus recommendations for the management of non-tuberculous mycobacteria in individuals with cystic fibrosis: executive summary. Thorax.

[15] Gilljam M, Schersten H, Silverborn M, Jonsson B, Ericsson Hollsing A (2010). Lung transplantation in patients with cystic fibrosis and Mycobacterium abscessus infection. J Cyst Fibros.

[16] Sanguinetti M, Ardito F, Fiscarelli E, La Sorda M, D’Argenio P, Ricciotti G, Fadda G (2001). Fatal pulmonary infection due to multidrug-resistant Mycobacterium abscessus in a patient with cystic fibrosis. J Clin Microbiol.

[17] Taylor JL, Palmer SM (2006). Mycobacterium abscessus chest wall and pulmonary infection in a cystic fibrosis lung transplant recipient. J Heart Lung Transplant.

[18] Chernenko SM, Humar A, Hutcheon M, Chow CW, Chaparro C, Keshavjee S, Singer LG (2006). Mycobacterium abscessus infections in lung transplant recipients: the international experience. J Heart Lung Transplant.

[19] Robinson PD, Harris KA, Aurora P, Hartley JC, Tsang V, Spencer H (2013). Paediatric lung transplant outcomes vary with Mycobacterium abscessus complex species. Eur Respir J.

[20] Huang HC, Weigt SS, Derhovanessian A, Palchevskiy V, Ardehali A, Saggar R, Saggar R, Kubak B, Gregson A, Ross DJ (2011). Non-tuberculous mycobacterium infection after lung transplantation is associated with increased mortality. J Heart Lung Transplant.

[21] Qvist T, Pressler T, Thomsen VO, Skov M, Iversen M, Katzenstein TL (2013). Nontuberculous mycobacterial disease is not a contraindication to lung transplantation in patients with cystic fibrosis: a retrospective analysis in a Danish patient population. Transplant Proc.

[22] Lobo LJ, Chang LC, Esther CR, Gilligan PH, Tulu Z, Noone PG (2013). Lung transplant outcomes in cystic fibrosis patients with pre-operative Mycobacterium abscessus respiratory infections. Clin Transpl.

[23] Chalermskulrat W, Sood N, Neuringer IP, Hecker TM, Chang L, Rivera MP, Paradowski LJ, Aris RM (2006). Non-tuberculous mycobacteria in end stage cystic fibrosis: implications for lung transplantation. Thorax.

[24] Weill D, Benden C, Corris PA, Dark JH, Davis RD, Keshavjee S, Lederer DJ, Mulligan MJ, Patterson GA, Singer LG (2015). A consensus document for the selection of lung transplant candidates: 2014--an update from the Pulmonary Transplantation Council of the International Society for Heart and Lung Transplantation. J Heart Lung Transplant.

[25] Aitken ML, Limaye A, Pottinger P, Whimbey E, Goss CH, Tonelli MR, Cangelosi GA, Dirac MA, Olivier KN, Brown-Elliott BA (2012). Respiratory outbreak of Mycobacterium abscessus subspecies massiliense in a lung transplant and cystic fibrosis center. Am J Respir Crit Care Med.

